# Supplementary material for: Dynamics of Antibiotic Resistance of Streptococcus pneumoniae in France: A Pediatric Prospective Nasopharyngeal Carriage Study from 2001 to 2022
Source: Antibiotics (Basel). 2023 Jun 6;12(6):1020. doi: 10.3390/antibiotics12061020 (PMC10295685; doi:10.3390/antibiotics12061020)
Supplement: Supplementary file 1 [file antibiotics-12-01020-s001.zip › antibiotics-2399187-supplementary.pdf]

Supplementary Table S1. Serotype distribution during the pre-PCV7, and PCV7 periods.

Periods	Pre-PCV7 period (N=671) September 2001 to December 2002		PCV7 period (N=1,987) June 2006 to May 2010	
	Total strains, n (%)	Penicillin non-susceptible strains, n (penicillin non-susceptibility rate)	Total strains, n (%)	Penicillin non-susceptible strains, n (penicillin non-susceptibility rate)
**PCV7 serotypes**				
19F	116 (17.3)	81 (69.8)	118 (5.9)	100 (84.7)
6B	103 (15.3)	79 (76.7)	29 (1.5)	14 (48.3)
23F	95 (14.2)	79 (83.1)	24 (1.2)	17 (70.8)
14	72 (10.7)	69 (95.8)	13 (0.6)	10 (76.9)
9V	21 (3.1)	20 (95.2)	4 (0.2)	4 (100)
18C	12 (1.8)	1 (8.3)	4 (0.2)	1 (25.0)
**Non-PCV7/PCV13+6C serotypes**				
19A	79 (11.8)	61 (77.2)	457 (23.0)	414 (90.6)
6A	62 (9.2)	20 (32.2)	74 (3.7)	18 (24.3)
3	15 (2.2)	0 (0)	66 (3.1)	1 (1.5)
6C	5 (0.7)	4 (80.0)	85 (4.3)	24 (28.2)
7F	1 (0.2)	0 (0)	38 (1.9)	0 (0)
**Non-PCV13/PCV15 serotypes**				
33F	4 (0.6)	4 (100.0)	22 (1.1)	0 (0)
22F	1 (0.1)	0 (0)	37 (1.9)	1 (2.7)
**Non-PCV15/PCV20 serotypes**				
15B/C	17 (2.5)	14 (82.3)	118 (5.9)	34 (28.8)
11A	12 (1.8)	0 (0)	97 (4.9)	6 (6.2)
10A	1 (0.1)	0 (0)	40 (2.0)	1 (2.5)
**Non-PCV20 serotypes**				
38	9 (1.2)	0 (0)	12 (0.6)	1 (8.3)
17F	5 (0.8)	1 (20.0)	37 (1.9)	3 (8.1)
21	5 (0.8)	0 (0)	46 (2.3)	1 (2.2)
23A	4 (0.6)	1 (75.0)	86 (4.3)	5 (5.8)
24F	4 (0.6)	4 (100)	42 (2.1)	17 (40.5)
15A	4 (0.6)	3 (75.0)	143 (7.2)	124 (86.7)
23B	1 (0.1)	1 (100)	61 (3.1)	6 (9.8)
35F	1 (0.1)	0 (0)	46 (2.3)	4 (8.7)
35B	0 (0)	0 (0)	112 (5.6)	99 (88.4)

Note: serotypes isolated in fewer than <1.0% of patients during both periods are not shown.

Supplementary Table S2. Serotype distribution during the early PCV13, and late PCV13 periods.

Periods	Early PCV13 period (N=1,983) June 2010 to April 2014		Late PCV13 period (N=1,987) May 2014 to July 2022	
	Total strains, n (%)	Penicillin non-susceptible strains, n (penicillin non-susceptibility rate)	Total strains, n (%)	Penicillin non-susceptible strains, n (penicillin non-susceptibility rate)
**PCV7 serotypes**				
19F	50 (2.5)	45 (90.0)	124 (3.3)	118 (95.2)
**Non-PCV7/PCV13+6C serotypes**				
19A	168 (8.5)	155 (92.3)	77 (2.0)	63 (81.8)
6C	99 (5.0)	33 (33.3)	40 (1.1)	4 (10.0)
3	31 (1.6)	0 (0)	45 (1.2)	0 (0.0)
**Non-PCV13/PCV15 serotypes**				
22F	47 (2.4)	2 (4.2)	84 (2.2)	4 (47.6)
33F	43 (2.2)	3 (7.0)	66 (1.7)	3 (4.5)
**Non-PCV15/PCV20 serotypes**				
15B/C	244 (12.3)	73 (29.9)	540 (14.3)	143 (26.5)
11A	180 (9.1)	46 (25.5)	363 (9.6)	252 (69.4)
10A	69 (3.5)	4 (5.8)	178 (4.7)	2 (1.1)
**Non-PCV20 serotypes**				
15A	184 (9.3)	163 (88.6)	277 (7.3)	200 (72.2)
35B	147 (7.4)	138 (93.9)	244 (6.5)	215 (88.1)
23A	120 (6.0)	6 (5.0)	216 (5.7)	69 (31.9)
24F	67 (3.4)	16 (23.9)	114 (3.0)	80 (70.2)
21	67 (3.4)	1 (1.5)	207 (5.5)	3 (1.4)
35F	59 (3.0)	2 (3.4)	193 (5.1)	4 (2.1)
17F	48 (2.4)	4 (8.3)	56 (1.5)	7 (12.5)
Non-typable	39 (2.0)	24 (61.5)	106 (2.8)	84 (79.2)
31	38 (1.9)	0 (0)	68 (1.8)	3 (4.4)
16F	23 (1.2)	1 (4.3)	67 (1.8)	6 (8.9)
29	14 (0.7)	14 (100)	40 (1.1)	36 (90.0)
9N	10 (0.5)	0 (0)	61 (1.6)	2 (3.3)

Note: serotypes isolated in fewer than <1.0% of patients during both periods are not shown.”.

Supplementary Table S3. Carriage and penicillin non-susceptibility of emerging serotypes identified during the late PCV13 period

<table>
<tr><th>Periods</th><th>Pre-PCV7</th><th>Targeted PCV7</th><th>PCV7</th><th>Early PCV13</th><th>Late PCV13</th></tr>
<tr><td>Carriage of serotype 15B/C</td><td colspan="5">+0.37% (0.24 to 0.50), p<0.001</td></tr>
<tr><td>Penicillin non-susceptibility of serotype 15B/C</td><td colspan="4">-0.90% (-1.23 to -0.57), p<0.001</td><td>+0.01% (+0.007 to +0.02), p<0.001</td></tr>
<tr><td>Carriage of serotype 23B</td><td colspan="5">+0.40% (+0.20 to +0.60), p<0.001</td></tr>
<tr><td>Penicillin non-susceptibility of serotype 23B</td><td colspan="4">NA</td><td>+0.40% (+0.21 to +0.57), p<0.001</td></tr>
<tr><td>Carriage of serotype 11A</td><td colspan="4">+0.46% (0.28 to +0.65), p<0.001</td><td>-0.03% (-0.003 to -0.05), p=0.02</td></tr>
<tr><td>Penicillin non-susceptibility of serotype 11A</td><td colspan="4">NA</td><td>+0.53% (+0.23 to +0.84), p<0.001</td></tr>
</table>

Note: the “pre-PCV7 period” was from September 2001 to December 2002, the “targeted PCV7 period” from January 2003 to May 2006, the “PCV7 period” from June 2006 to May 2010, the “early PCV13 period” from June 2010 to April 2014, the “late PCV13 period” from May 2014 to July 2022.

NA: model not available because of the very low number of cases.

Supplementary Table S4. Factors affecting carriage of antibiotic non-susceptible *S. pneumoniae* by univariate and multivariate analysis during the late PCV13 period.

	**Univariate analysis**			**Multivariate analysis**		
	Odds ratio	95% confidence interval	P-value	Adjusted odds ratio	95% confidence interval	P-value
**Carriage of penicillin non-susceptible *S. pneumoniae*** N=3,776						
Age <1 year	0.99	0.87 to 1.13	0.87			
Siblings	0.87	0.76 to 1.01	0.05			
Recent use of antibiotics	1.89	1.65 to 2.17	<0.001	1.56	1.28 to 1.88	<0.001
Recent use of broad antibiotics	1.64	1.33 to 2.02	<0.001			
Daycare center	1.37	1.20 to 1.58	<0.001	1.25	1.07 to 1.47	0.006
Fever (≥38.5°C)	0.86	0.75 to 0.99	0.03			
Otalgia	1.07	0.93 to 1.24	0.30			
History of AOM	1.81	1.55 to 2.10	<0.001	1.36	1.13 to 1.64	0.001
Otitis-prone children	1.49	1.24 to 1.80	<0.001			
Otorrhea	0.88	0.69 to 1.12	0.31			
Carriage of Hi	1.17	1.02 to 1.34	0.02			
Conjunctivitis	1.16	0.99 to 1.37	0.06			
Bilateral AOM	1.01	0.88 to 1.15	0.87			
**Carriage of erythromycin non-susceptible *S. pneumoniae***						
Age <1 year	1.02	0.88 to 1.18	0.77			
Siblings	0.92	0.79 to 1.06	0.25			
Recent use of antibiotics	1.53	1.31 to 1.78	<0.001	1.36	1.10 to 1.68	0.004
Recent use of broad antibiotics	1.60	1.28 to 2.00	<0.001			
Daycare center	1.23	1.05 to 1.43	0.009			
Fever (≥38.5°C)	0.90	0.78 to 1.05	0.17			
Otalgia	0.94	0.80 to 1.10	0.44			
History of AOM	1.51	1.27 to 1.78	<0.001	1.27	1.03 to 1.56	0.02
Otitis-prone children	1.15	0.94 to 1.41	0.17			
Otorrhea	0.85	0.65 to 1.13	0.27			
Carriage of Sp	1.05	0.91 to 1.22	0.48			
Conjunctivitis	1.14	0.96 to 1.37	0.14			
Bilateral AOM	0.92	0.79 to 1.06	0.26			

Note: the “late PCV13 period” was from May 2014 to July 2022.

Supplementary Table S5. Estimation of the impact of non-pharmaceutical intervention implementation on carriage and antibiotic resistance of *Streptococcus pneumoniae*

	**Model with an immediate change and a trend during the COVID-19 period**			
	Immediate change (95% CI)	P-value	Monthly trend (95% CI)	P-value
**Overall Sp carriage**	-18.1% (-46.1 to +9.8)	0.20	+0.45% (-0.46 to 1.37)	0.32
**Rate of penicillin non-susceptible Sp strains**	+10.6% (-27.6 to 48.7)	0.58	-0.31% (-2.47 to +1.85)	0.77
**Rate of erythromycin non-susceptible Sp strains**	+12.2% (-49.5 to +73.9)	0.70	-0.39% (-1.54 to +0.76)	0.49

Note: estimations are based on the model using segmental linear regression with autoregressive error, COVID-19 period was from April 2020 to July 2022

Abbreviations: CI, confidence interval

Supplementary Figure S1. Evolution of antibiotic use in the 3 months before enrollment, and proportion of broad antibiotic among the children enrolled in the study.

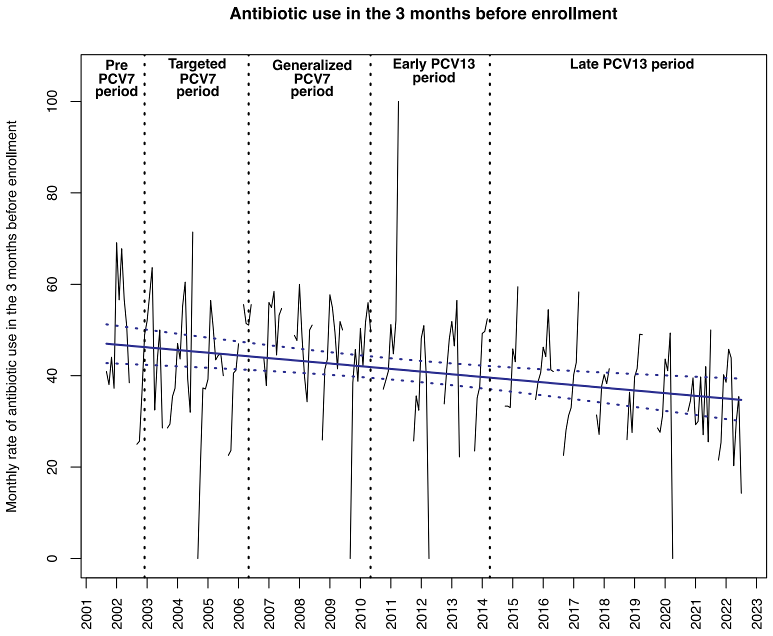

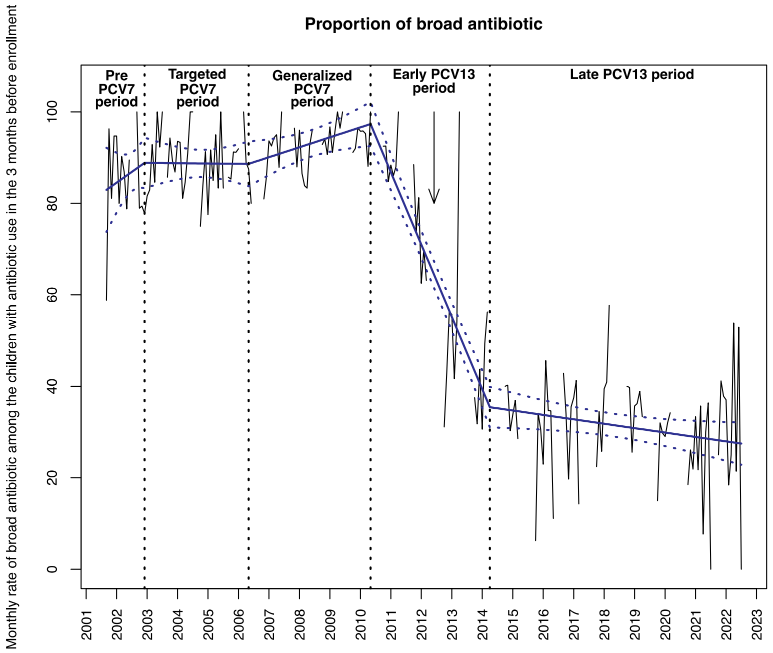

Notes: the black lines show the observed data. Using a segmented regression model, we have estimated the rates over time (blue slope lines) and its 95% confidence interval (blue dotted lines). The "pre-PCV7 period" was from September 2001 to December 2002, the "targeted PCV7 period" from January 2003 to May 2006, the "PCV7 period" from June 2006 to May 2010, the "early PCV13 period" from June 2010 to April 2014, the "late PCV13 period" from May 2014 to July 2022. The black arrow shows the recommendation to use amoxicillin as first-line antibiotic for acute otitis media in children.

Supplementary Figure S2. Evolution of carriage of serotype 15B/C and penicillin non-susceptibility among the isolated strains.

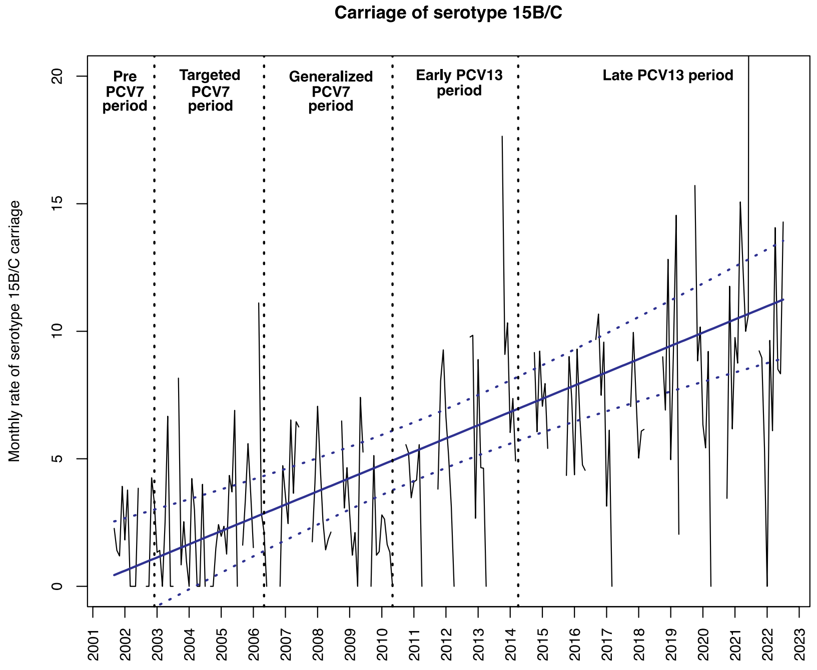

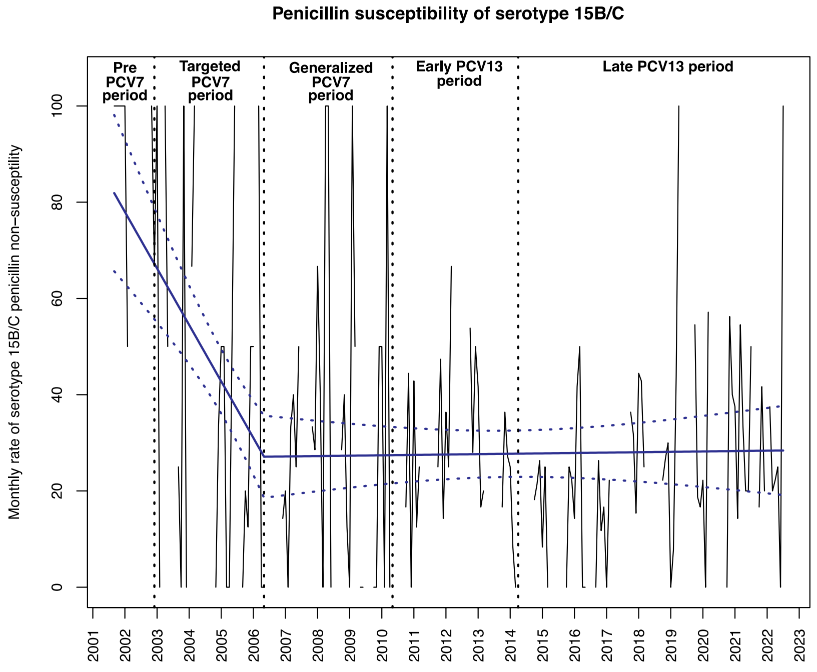

Notes: the black line shows the observed data. Using a segmented regression model, we have estimated the rates over time (blue slope lines) and its 95% confidence interval (blue dotted lines). The "pre-PCV7 period" was from September 2001 to December 2002, the "targeted PCV7 period" from January 2003 to May 2006, the "PCV7 period" from June 2006 to May 2010, the "early PCV13 period" from June 2010 to April 2014, the "late PCV13 period" from May 2014 to July 2022.

Supplementary Figure S3. Evolution of carriage of serotype 23B and penicillin non-susceptibility among the isolated strains.

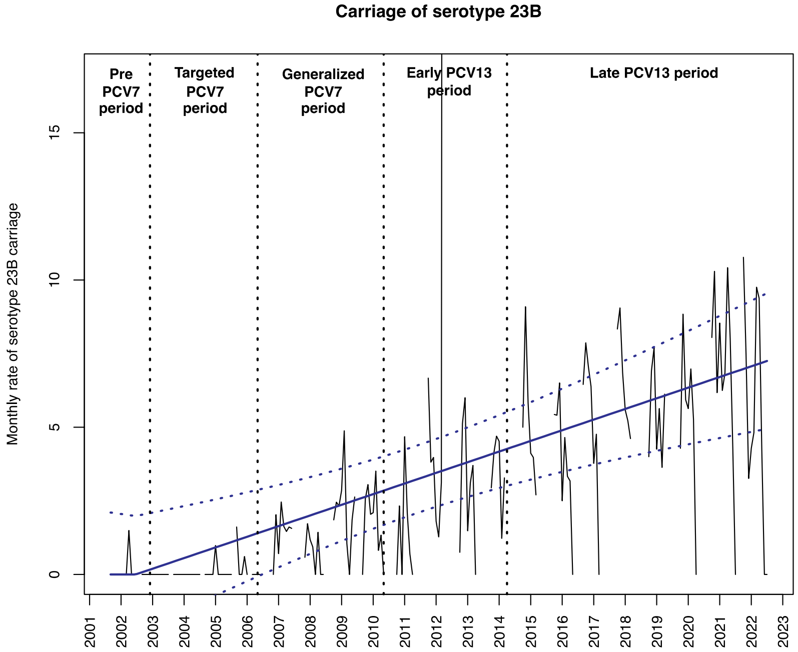

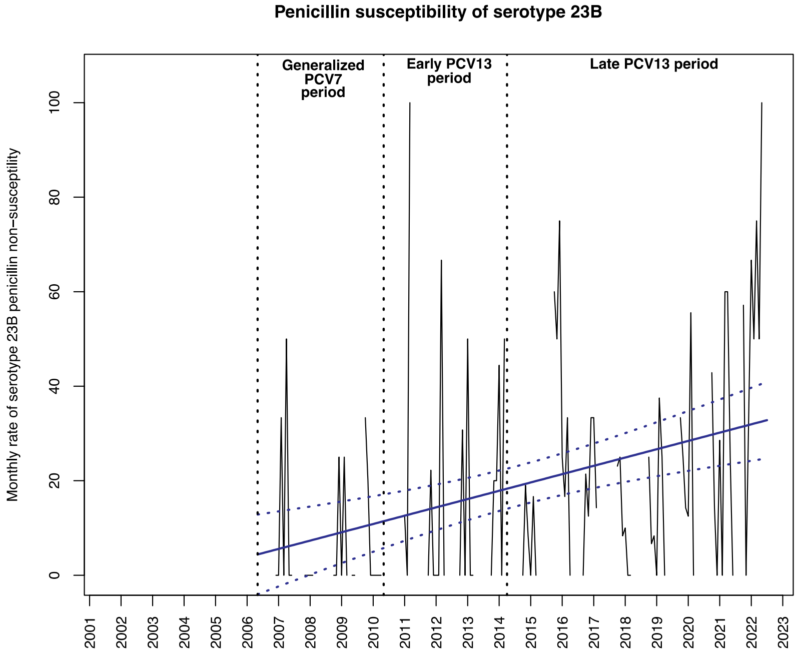

Notes: the black line shows the observed data. Using a segmented regression model, we have estimated the rates over time (blue slope lines) and its 95% confidence interval (blue dotted lines). The "pre-PCV7 period" was from September 2001 to December 2002, the "targeted PCV7 period" from January 2003 to May 2006, the "PCV7 period" from June 2006 to May 2010, the "early PCV13 period" from June 2010 to April 2014, the "late PCV13 period" from May 2014 to July 2022.

Supplementary Figure S4. Evolution of carriage of serotype 11A and penicillin non-susceptibility among the isolated strains.

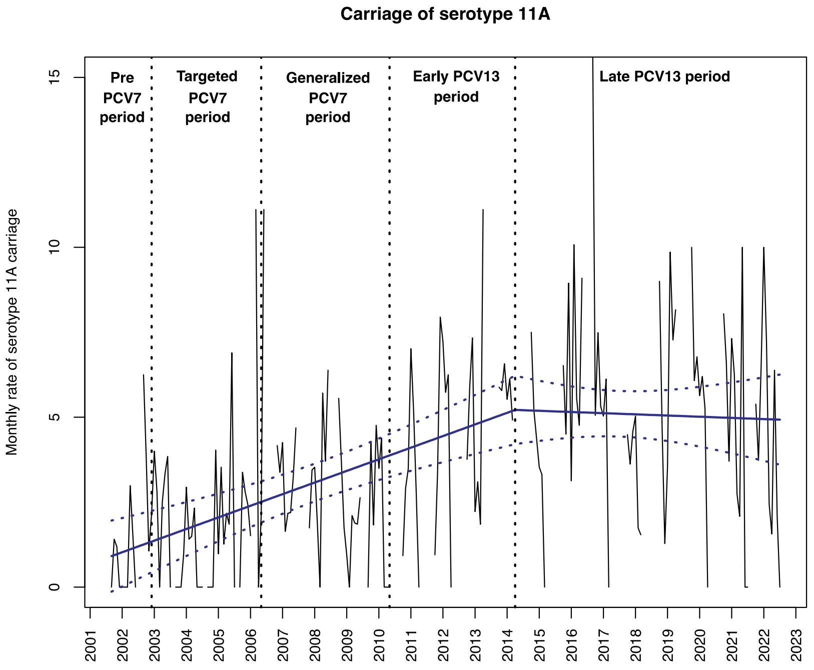

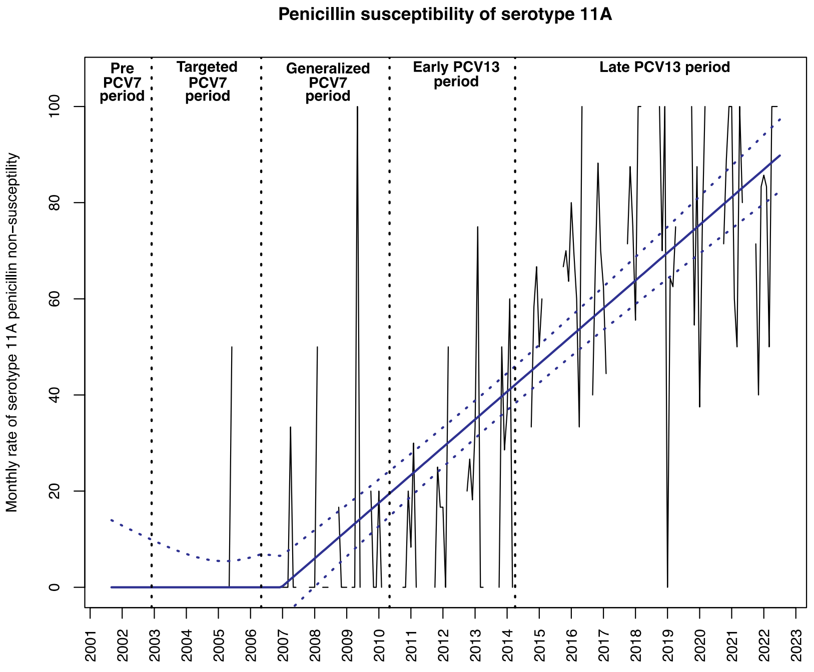

Notes: the black line shows the observed data. Using a segmented regression model, we have estimated the rates over time (blue slope lines) and its 95% confidence interval (blue dotted lines). The “pre-PCV7 period” was from September 2001 to December 2002, the “targeted PCV7 period” from January 2003 to May 2006, the “PCV7 period” from June 2006 to May 2010, the “early PCV13 period” from June 2010 to April 2014, the “late PCV13 period” from May 2014 to July 2022.
